# Revisiting radial forearm free flap for successful venous drainage

**DOI:** 10.1186/s40902-017-0110-8

**Published:** 2017-05-25

**Authors:** Yong Hoon Cha, Woong Nam, In-Ho Cha, Hyung Jun Kim

**Affiliations:** Department of Oral and Maxillofacial Surgery, Yonsei University College of Dentistry, 708 Yonsei University Dental Hospital 50-1 Yonsei-ro, Seodaemoon-gu Seoul, 03722 South Korea

**Keywords:** Radial forearm free flap, Venae comitantes, Coalesced vein

## Abstract

Tissue defect reconstruction using radial forearm free flap (RFFF) is a common surgical technique whose success or failure is mainly dependent on venous drainage. RFFF has two major venous outflow systems, superficial and deep vein. Drainage methods include combining both systems or using one alone. This review aims to recapitulate the vascular anatomy and network of RFFF as well as shed light on deep vein as a reliable venous drainage system. We also discuss basic evidence for and advantages of single microanastomosis with coalesced vein to overcome technical difficulties associated with the deep vein system.

## Introduction

Radial forearm free flap (RFFF) has been a workhorse in head and neck reconstruction since the first report of its usage in releasing scar contracture of burned patients [1, 2]. Several prefabricating methods were developed after knowledge of the so-called Chinese flap spread to the west [1, 3–6]. In particular, RFFF is applied in oral cavity reconstruction such as tongue [7], cleft lip, and palate rehabilitation [8, 9], as well as various defects originated from oral cavity cancer ablation surgery. By including the bony segment of the radius, an osteocutaneous flap can be raised, which might be proposed for mandible reconstruction [4, 10]. Moreover, RFFF can cover most of the oral cavity by combining the medial and lateral cutaneous nerves with the tendon of the palmaris longus muscle [11–13].

RFFF offers ease of harvesting and reliability due to its constant, reproducible vascular anatomy. It is also versatile owing to the relatively long pedicle and thin, pliable, hairless skin paddle. A two-team approach is possible in head and neck surgery, various forms being applicable, for example, free or pedicled, proximally or distally. However, functional, esthetic issues and donor site morbidity are inevitable complications necessitating local rotation and advancement flap or skin grafting, while primary closure is only possible with a cutaneous flap less than 2 ~ 3 cm. Both split and full-thickness skin grafts are possible, but the latter ensure the better cosmetic appearance. Osteofasciocutaneous flap accompanying radius needing a subsequent 6-week immobilization can be harvested for mandible reconstruction, but its usage is limited due to the high risk of fracture from low mechanical strength. The most important aspect of the RFFF harvesting procedure is confirmation of hand vascularity requiring at least two independent Allen tests or angiography to check the recovery of superficial palmar arch by the ulnar artery [14, 15].

Although RFFF yields a dependable success rate, the most common reason for failure is inadequate venous drainage [16, 17]. RFFF has two major venous drainage systems, superficial and deep vein, the former utilizing the cephalic vein and the latter the venae comitantes. Given the relatively lower flow pressure of venous drainage compared to the arterial stream, veins are easily obstructed by extrinsic compression and thrombi can be generated by slight intimal damage. Technical countermeasures to thrombus formation include reducing pedicle tension and kinking as well as medical treatment to decrease vascular spasm. Nowadays, such efforts have reduced the failure rate of free flap transplantation from 17 to 4% [18]. However, anastomosis of venae comitantes remains technically challenging for surgeons, particularly for beginners. This review thus recapitulates the basic anatomy and vascular network of RFFF to suggest methods for accomplishing better venous drainage.

## Review

### Vascular anatomy of RFFF

The main feeding vessel of RFFF, the radial artery, originates from the brachial artery at around 2 cm distal of the elbow, where the ulnar artery is also divided. The radial artery runs between the brachioradialis and pronator teres muscles at the proximal third, and follows the lateral intermuscular septum between the brachioradialis and flexor carpi radialis at the distal. The wrist area becomes a surgical landmark offering palpable pulsation of the radial artery as there is no muscle coverage. Crucially, the radial artery in the fasciocutaneous flap provides numerous branches to overlying subcutaneous tissue, skin, flexor muscles, and underlying periosteum of distal radius through deep fascia. The approximately five to seven perforating arteries arising from the radial artery at the lower arm constitute the arterial inflow of the radial forearm fasciocutaneous paddle. It is thus critical to closely attach the skin and the subcutaneous fascial layer during flap harvesting. Also, a strip of skin at least 3 cm width overlying the posterior extensor compartment of the forearm and the ulnar subcutaneous border should be kept intact due to poor vascularity of the radial artery in this region. The usual pedicle length of the radial artery is about 18 cm and the width of lumen is around 3 mm, which offers the proper length and size to perform microanastomosis in the head and neck region with facial, superior thyroidal, and superficial temporal arteries.

The radial artery always accompanies two venae comitantes which communicate with each other in a ladder shape. Deep venae comitantes drain into the median cubital vein, communicating with superficial veins at the elbow area. The cephalic vein is the most commonly used single vein for venous drainage of RFFF. It is a large, fairly thick-walled vein found in a relatively constant location deep beneath the subcutaneous fat. Due to its size and superficial position, it is also very often used for intravenous lines, which may cause fibrosis and/or thrombosis of the vessel. It drains the anterolateral forearm and is formed mainly by the confluence of superficial veins on the dorsal aspect of the hand. From there, the vein, or its tributaries, traverses the lateral snuffbox area to lie over the lateral side of the distal forearm. It gradually courses more medially towards the midlateral cubital fossa. The lumen width of the venae comitantes is around 1.5 mm, while the cephalic vein shows 3 mm or more. Extremely narrowed lumens are observed with notable frequency in venae comitantes contrary to the cephalic vein. The difficulties of anastomosing venae comitantes mainly determine the survival of RFFF although the process is becoming easier. Valves manifest more frequently on deep veins than on superficial veins [19], but the frequent interconnection permits bypassing and retrograde flow, which support the distally pedicled flap [20–23].

### Vascular network of fasciocutaneous flap

Advocates of the single superficial vein drainage system emphasize ease of harvesting and microanastomosis while noting it is not inferior to the deep alone or dual systems [24, 25]. However, the most common cause of flap failure is venous thrombus originated from a superficial vein system [17]; moreover, cephalic vein occlusion due to previous intravenous cannulation causes failure of RFFF despite apparent normalcy during flap harvesting [26]. In addition to these vascular problems, a dorsally extended skin flap design accompanying cephalic vein results in less effective compression of postoperative tie-over dressing than the standard volar surfaced flap design. Hence, the reliability of the superficial alone system remains controversial [27].

**Fig. 1:**
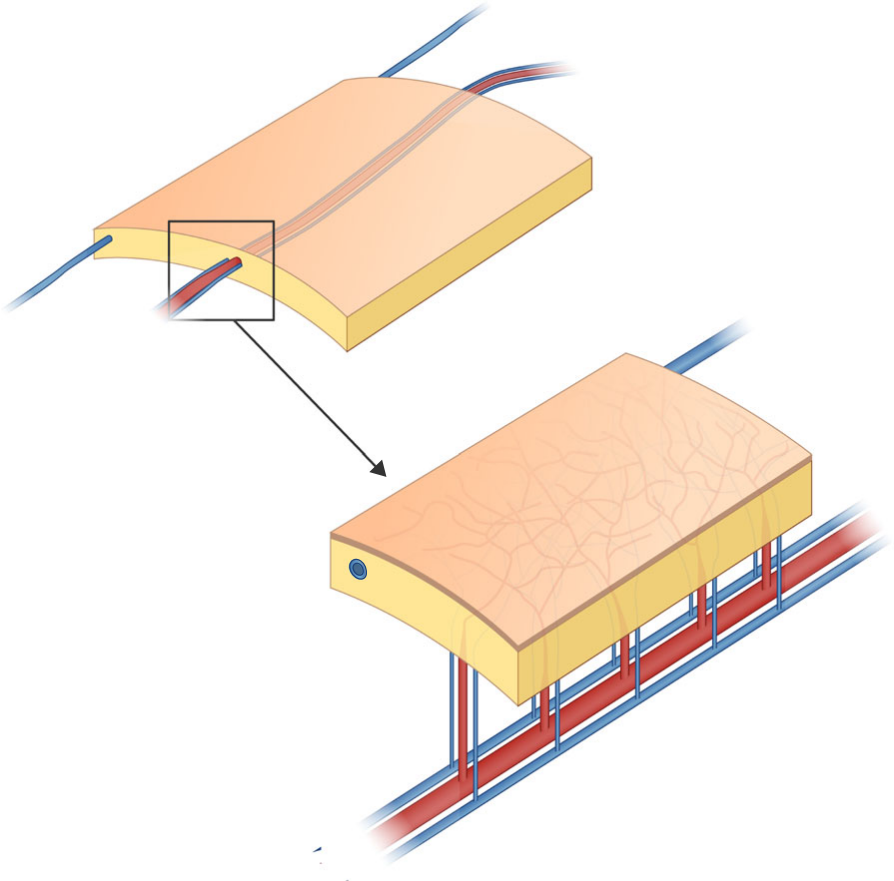
Schematic structure of the fasciocutaneous flap. Please note the septocutaneous perforator and complex vascular network originating from radial artery and venae comitantes. The cephalic vein passes through the subcutaneous layer without sprouting branches

A hemodynamic study demonstrated that the deep veins have twice the volume of drainage per unit time compared with the superficial vein [28, 29]. Furthermore, Demirkan et al. reported no venous compromise or partial/complete flap loss throughout the study of 94 consecutive RFFFs using a single venae comitantes anastomosis [30]. The fundamental vascular network of fasciocutaneous flap is made up of numerous invisible arterial/venous communications within the fascial layer, the so-called septocutaneous vascular network, which arises from arterial perforators and accompanies venae comitantes (Fig. 1). In contrast, superficial veins simply pass through the subcutaneous layer. Although surgeons tend to select the superficial system as an alternative or additional option in RFFF due to difficulties in anastomosing venae comitantes, the primary choice of venous drainage in RFFF is mostly dependent on hemodynamic knowledge of the deep vein system.

### Raising RFFF with a single large deep vein system

**Fig. 2:**
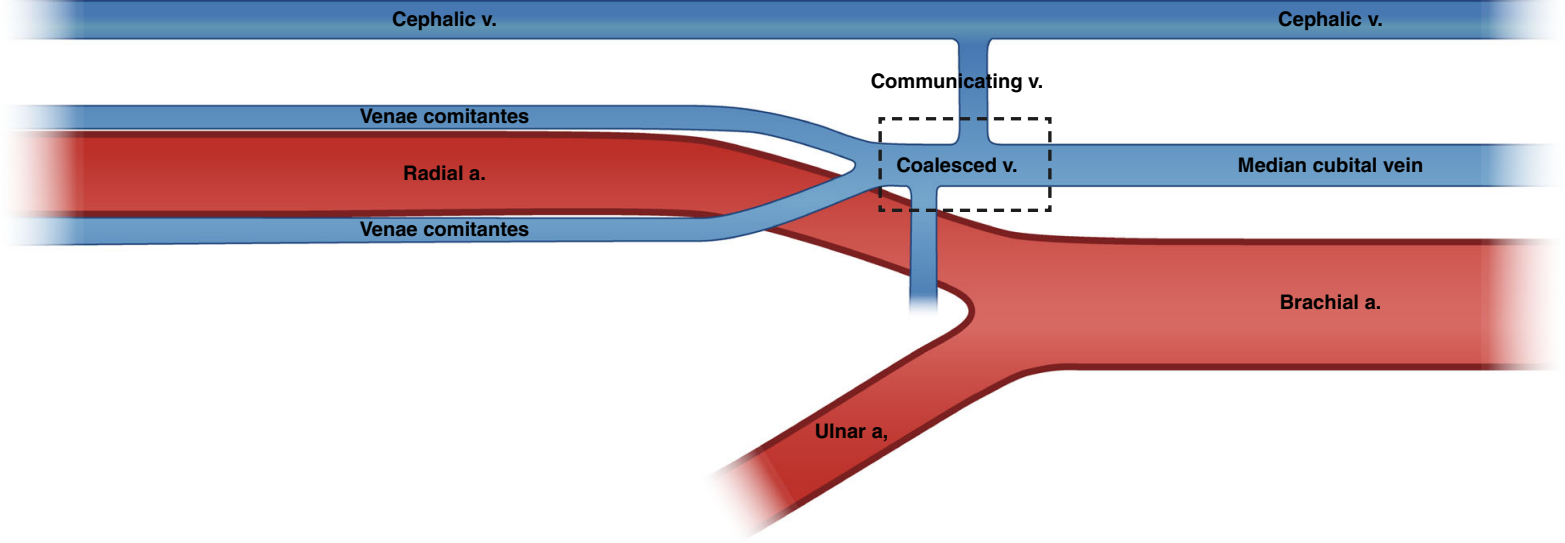
Schematic vascular structure of RFFF. Two venae comitantes are anastomosed at the coalesced vein, which drains into the median cubital vein

Both venae comitantes are confluenced at the proximal end of the radial artery around the bifurcation point of the brachial artery. There, those two small deep veins are joined into a single, short larger vein, the coalesced vein, just before draining into the medial cubital vein (Fig. 2). The length of coalesced vein is about 0.25~1.5 cm. If the surgeon cuts the pedicle at the distal point of the coalesced vein, one artery and one or two venae comitantes should be anastomosed. However, if vein cutting and vessel preparation is done at the proximal of the coalesced vein, microanastomosis is accomplished with one artery and one larger vein. In the case of the combined superficial and deep venous drainage system, the profundus cubitalis vein connects the coalesced vein to the cephalic vein at the level of the cubital fossa although its anatomical consistency is unreliable [31, 32]. The main advantage of the coalesced vein is its lumen width being almost that of the cephalic vein [19].

Surgical considerations for harvesting the colaesced vein in RFFF are as follows. First, dissection should be extended to the antecubital fossa area. Second, the venous anatomy of the antecubital fossa is complex. However, as only the coalesced vein needs to be clamped in microanastomosis, the elongation and extended dissection are not required. When using the coalesced vein in RFFF, sufficient venous drainage from the hemodynamically superior deep vein system as well as a satisfactory lumen width for easy vein anastomosis (shortening operation time) are required. Moreover, flap versatility is increased by a lengthened pedicle. A study of venous variants in 40 consecutive RFFFs reported 80% of flaps being harvested at the coalesced vein or more proximal veins with successful results [32]. Intriguingly, cases of anastomosing both venae comitantes were less than 5%, 15% showing successful results using one of the venae comitantes.

If a longer and larger vein than the coalesced vein is needed, particularly in the case of anastomosis on the contralateral neck, a proximal dissection extension to the median cubital or basilic vein can be performed. However, excessive length causes kinking or twisting of the pedicle, which threatens survival of the free flap. Thus, pedicle harvesting at the coalesced vein is sufficient in usual oral cavity reconstruction to achieve stable venous drainage and easy microanastomosis. If an undesirably long pedicle is anticipated, a proximal skin paddle design can be considered. Also, considering that skin paddle thickness gradually increases from distal to proximal in the volar aspect of lower arm, thin subcutaneous fascial layer harvesting with preserving sufficient paratendon and myofascia is recommended.

## Conclusions

Controversy as to the better vein system in RFFF has arisen since the technique’s emergence. Initially, the combined vein system was recommended although the superficial system was regarded as primary [2]. A series of studies revealed failure of the superficial alone system due to flap edema and congestion during penile reconstruction [33]. In this regard, venae comitantes were deemed more favorable [18, 34]. Furthermore, owing to the investigation of RFFF failure caused by venous thrombus within superficial veins, the deep vein system has become the preferred method [17]. Hence, we reviewed the vascular anatomy and network of RFFF to revisit fundamental principles and suggested the coalesced vein, which is a single large confluence deep venous drainage system, as a favorable choice for young oral and maxillofacial surgeons aiming to perform easy and safe tissue transfer surgery.
